# Podosomes and Invadopodia: Related structures with Common Protein Components that May Promote Breast Cancer Cellular Invasion

**DOI:** 10.4137/bcbcr.s789

**Published:** 2008-05-29

**Authors:** Daniel C. Flynn, YoungJin Cho, Deanne Vincent, Jess M. Cunnick

**Affiliations:** 1 Mary Babb Randolph Cancer Center; 2 Department of Microbiology, Immunology and Cell Biology and; 3 Department of Pathology, West Virginia University, Morgantown, WV 26506-9300

**Keywords:** invadopodia, podosomes, invasion, breast cancer

## Abstract

A rate-limiting step in breast cancer progression is acquisition of the invasive phenotype, which can precede metastasis. Expression of cell-surface proteases at the leading edge of a migrating cell provides cells with a mechanism to cross tissue barriers. A newly appreciated mechanism that may be relevant for breast cancer cell invasion is the formation of invadopodia, well-defined structures that project from the ventral membrane and promote degradation of the extracellular matrix, allowing the cell to cross a tissue barrier. Recently, there has been some controversy and discussion as to whether invadopodia, which are associated with carcinoma cells, are related to a similar structure called podosomes, which are associated with normal cells. Invadopodia and podosomes share many common characteristics, including a similar size, shape, subcellular localization and an ability to promote invasion. These two structures also share many common protein components, which we outline herein. It has been speculated that podosomes may be precursors to invadopodia and by extension both structures may be relevant to cancer cell invasion. Here, we compare and contrast the protein components of invadopodia and podosomes and discuss a potential role for these proteins and the evidence that supports a role for invadopodia and podosomes in breast cancer invasion.

## Introduction

Breast cancer is a complex disease that is estimated to affect 182,460 women in 2008 with 40,480 predicted mortalities in the United States, alone. The most commonly diagnosed form of breast cancer is invasive ductal carcinoma, which is usually detected as a stage I disease. When treated with standard therapy (lumpectomy, radiation and tamoxifen) invasive ductal carcinoma has a five-year survival rate of approximately 80%. Initially, invasive ductal carcinoma begins as an atypical hyperplasia, typified by a loss of balance between growth and apoptosis of the epithelial cells that line the breast ducts. Here, the cells appear to fill the duct and show a characteristic pattern of increased mitotic activity throughout the hyperplasia. The disease can then progress to ductal carcinoma in situ where it remains contained within the ducts; however, mitotic activity is elevated throughout the tumor. Subsequently, these cells can become invasive. They can move as either a collective “sheet” of cells or they can separate away from the ductal carcinoma in situ and move independently. These newly invasive cells can breach the barrier of the ducts and move into the collagen matrix of the breast where they can establish a tumor. Invasion requires increased migratory capacity and protease expression. Ultimately, these cells may gain entry into the lymph nodes where they can metastasize, or they may intravasate directly into blood vessels, where they can be transported and trapped within the capillaries. Here, the cells can extravasate into surrounding tissue and potentially establish a distant site metastasis. Thus, a key feature in the progression of breast cancer is acquisition of the invasive phenotype. Clearly, if breast cancer invasion could be blocked, tumor growth would be confined and the disease rendered manageable.

Invasion occurs by different mechanisms. Migrating cells may express and secrete proteases at the leading edge of the carcinoma cell. These proteases degrade the extracellular matrix (ECM) and create a path of least resistance through which cells migrate and cross tissue barriers (Gimona et al. 2008). Alternatively, carcinoma cells can ‘push’ their way through a loose matrix, moving in a fashion that might be analogous to amoeboid motility, which can occur independent of protease activity (Sahai and Marshall, 2003). Invasive cells can also move ventrally, using podosomes or invadopodia, both of which promote the local release of protease activity and allow the cell to degrade the extracellular matrix and cross a tissue barrier.

## Invadopodia and Podosomes

Invadopodia share many characteristics with podosomes, thus, there has been some controversy as to whether podosomes and invadopodia are related or distinct structures. Several very fine reviews have been written recently on this subject (Ayala et al. 2006; Yamaguchi and Condeelis, 2007; Linder, 2007; Gimona et al. 2008), that outline podosome and invadopodium structure and function and discuss some of the aspects of them that are common and distinct. At the core of this controversy is whether podosomes are precursors to invadopodia, and by extension, whether podosomes (like invadopodia) are relevant for cancer cell invasion. Alternatively, it has been speculated that podosomes and invadopodia could have both evolved from some common primordial structure. Here, we will review the protein components of podosomes and invadopodia and the data that indicate these structures may be related and relevant for breast cancer invasion.

## Structural Features

Both podosomes and invadopodia are functional structures that form on the ventral membrane of cells and modulate the release and activation of proteases that degrade the extracellular matrix and promote the ability of cells to cross tissue barriers. The main differences are the types of cells in which they have been identified and their relative size. Podosomes are associated with normal cells, such as macrophages, osteoclasts, dendritic cells, epithelial cells, smooth muscle cells and fibroblasts. They are relatively small, about 1.0 μm in diameter and extend into the matrix 0.5 μm in length (Linder and Aepfelbacher, 2003). Podosomes can coalesce and form larger, ‘donut’ shaped structures that appear to be clusters of podosomes and are about 5 μm in diameter (Gringel et al. 2006; Gu et al. 2007). This difference in size could be related to changes in higher order structure or could correlate in part with a difference in the organization of actin filaments within them (Gimona et al. 2008). Interestingly, the size of the structure appears to correlate with half-life. Podosomes have a relatively short half-life, 2–10 minutes, however, larger podosomes appear to have a longer half-life (Gringel et al. 2006; Gu et al. 2007). In another level of higher organization, podosomes can cluster and form a larger ring structure called a rosette, which is characteristic of oncogene-transformed fibroblasts (Linder and Aepfelbacher, 2003). In yet a third higher order structure, podosomes can cluster together like a tightly connected rosette and form a ‘sealing zone’, which is a characteristic structure associated with osteoclasts and their bone resorption function.

**Figure 1.**
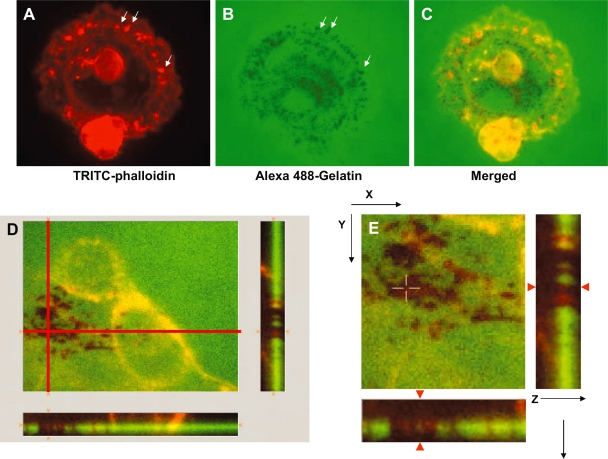
Invadopodia formation in Src527F-expressing MDA-MB-231 breast carcinoma cells. (**A**) TRITC-phalloidin labeling of F-actin demonstrates actin-rich punctate structures around the cell peripheray (arrows) as detected by confocal immunofluorescence microscopy on the ventral membrane (0.7 μm scanning thickness). (**B**) The cells were plated on Alexa488-gelatin/fibronectin and allowed to degrade the extracellular matrix, as seen by zones of clearing in the ‘green’ extracellular matrix (arrows). (**C**) Merged image shows the overlap of F-actin with proteolytic activity. (**D**) Larger panel shows zones of clearing or active proteolysis. The rectangular images below and beneath illustrate a cross section of degraded extracellular matrix showing ‘red’ F-actin protruding into the ‘green’ extracellular matrix by both x-z and y-z images (note where the red lines intersect, cells are turned on the side and ‘red’ actin is detected in the zones of clearing which now lack ‘green’ extracellular matrix. (**E**) Close-up view of (**D**) where red arrow in x-z and y-z show ‘red’ F-actin protruding into the ‘green’ extracellular matrix as an invadopodia. Similarly, the rectangular images show ‘red’ actin present in zones of clearing where the ‘green’ extracellular matrix has been degraded. Cells were the kind gift of Susette Mueller (Bowden et al. 2006).

Invadopodia on the other hand are associated with carcinoma cells and have been described as larger structures, up to 8 μm in diameter and 2–5 μm in length based on immunofluorescence confocal microscopy analysis (Linder, 2007). Invadopodia can be detected, in part, by identifying F-actin in a structure of the appropriate size and shape, on the ventral membrane, using scanning confocal immunofluorescence microscopy (0.7 μm scanning thickness) (Fig. 1A-C). Herein, one can turn the cells on their side and detect the F-actin protruding into the extracellular matrix, which becomes degraded (no ‘green’) (Fig. 1D-E).

Interestingly, an electron microscopy ultrastructure study demonstrated that invadopodia had a slender structure, 0.8 μm–1.0 μm in diameter and 2 μm or more in length (Artym et al. 2006). However, another very thorough study by Buccione and colleagues using a combined electron microscopy and confocal light and immunofluorescence microscopy approach that appeared to show invadopodia can cluster together, which would make them appear as larger diameter structures by light microscopy (Baldassarre et al. 2003). This observation could be analogous to the difference between small and large podosomes (Gu et al. 2007). Invadopodia have a longer half-life than podosomes, estimated anywhere from 1–3 hours (Artym et al. 2006; Linder, 2007). However, invadopodia life span appears to correlate well with whether the carcinoma cell is migrating. Migrating cells showed shorter-lived invadopodia while static cells showed longer-lived invadopodia (Yamaguchi et al. 2005; Artym et al. 2006). It is not clear whether invadopodia formed in migrating cells have a different diameter relative to those that would form in a static cell. Nevertheless, it may be possible that carcinoma cells need to become static or less migratory (i.e. confront a tissue barrier) in order to generate a long-lived invadopodia.

## Protein Biomarkers for Podosomes and Invadopodia

Recently, a meeting on podosomes and invadopodia was held at Cold Spring Harbor where the relationship of podosomes and invadopodia were discussed (“Podosomes and Invadopodia: Signatures of the wandering cell?”, November 26–29, 2007, John Condeelis, Ph.D., Chair). Although it was not resolved whether these are related or distinct structures, it was generally agreed that there should be a set of criteria used to define an ‘actin-rich dot’ on a ventral cell membrane as a podosome or an invadopodia. The consensus suggestion was that these structures should be imaged on the ventral membrane by confocal immunofluorescence microscopy at a scanning thickness of 0.7–1.0 μm. These structures should express actin in the core, as well as a reliable marker protein that differentiates invadopodia and podosomes from focal adhesions, such as cortactin, Tks5 or dynamin (Linder, 2007). Lastly, the ‘biomarkers’ should be detected in association with functional proteolytic activity by seeding the cells on a FITC-gelatin/fibronectin matrix and demonstrating that the biomarker for podosomes or invadopodia appear over zones of clearing where proteases have digested the matrix (Bowden et al. 2001). Using these criteria, a number of proteins have been described as associated with podosomes and invadopodia (Table 1). As podosomes are better studied than invadopodia, more protein components have been identified in association with podosomes. Nevertheless, it is clear that they share at least 32 common protein components, and likely more. In only one case did we find a controversy where one protein, tubulin, may be uniquely relevant for podosomes. Microtubular structures appear to be required for podosome dynamics but may be less important for invadopodia (Linder et al. 2000; Destaing et al. 2003; Destaing et al. 2005). In agreement with a role for microtubular structures supporting podosome dynamics, treatment of osteoclast cells with nocodazole did disrupt podosome location (Babb et al. 1997) and the microtubular-associated protein kinesin appears to be important for podosome dynamics (Kopp et al. 2006). Although there are data to indicate tubulin could be associated with invadopodia (Strohmaier et al. 2000), treatment of the Met-1 breast cancer cell line with colchicine did not inhibit invadopodia formation (Bourguignon et al. 1998). In this regard, it has been speculated that because podosomes are more dynamic structures than invadopodia, microtubules may not be required for invadopodia formation and function (Linder, 2007). If true, then it would be interesting to determine if there is a differential requirement of microtubular structures for larger, long-lived podosomes relative to smaller, short-lived podosomes. This is an understudied area that warrants a closer look. Otherwise, the protein components of podosomes and invadopodia listed in Table 1 tell a similar story. Actin filaments form the core of theses structures and an array of actin filament contractility, cross linking, branching and severing/capping proteins are represented in each structure and regulate the dynamic changes in actin filament organization, and by extension, the shape and the half-life of these structures in response to cellular signals. There are also proteins in place that can link the cytoskeleton to integrins and/or the membrane, which would promote interactions with the extra-cellular matrix. Adaptor proteins are present, which could serve to bridge interactions between signaling proteins such as tyrosine and serine kinases or phosphatases with the cytoskeleton are prevalent. These signaling proteins are predicted to regulate the architecture of these dynamic structures.

## Vesicle Transport and Podosome/Invadopodia Formation

Both podosomes and invadopodia contain small GTP binding proteins and regulatory proteins that that control their function. Within this class of proteins, dynamin and endophilin stand out as proteins that could bridge interactions of GTP binding proteins with membranes and promote the formation of a secretory canaliculi or the docking of vesicle membranes. Lastly, a variety of proteases are apparent, and most of them have been detected in invadopodia. To this end it is noteworthy that in invadopodia, TIMP-2 is able to block protease activity whereas TIMP-1 was not, indicating that invadopodia are more dependent upon membrane bound proteases than secreted proteases (Chen and Wang, 1999). Interestingly, both podosomes and invadopodia formation may require exocytosis, as brefeldin A and Exo 1 will block the formation of invadopodia and podosomes (Ayala et al. 2006; Walker et al. 2007). In this regard, it is also noteworthy that several proteins found associated with podosomes or invadopodia are normally associated with perinuclear vesicles in quiescent, normal cells, including cSrc, cortactin, Pyk2, dynamin 2, ADAM12, MT1-MMP and Tks5 (Kaplan et al. 1992; Redmond et al. 1992; Howell and Cooper, 1994; Fincham et al. 1996; Nicoziani et al. 2000; Hougaard et al. 2000; Kang et al. 2001). Thus, we would speculate that when cells make a decision to form a podosome or an invadopodia, outside-in signals could stimulate the movement of vesicles to the ventral membrane which in turn would deliver ‘cargo’ or protein components necessary for the formation of these structures. As podosomes and invadopodia will form rapidly, in less than 15 minutes after treatment with phorbol esters, and further, the formation of these structures do not require *de novo* protein synthesis (Linder and Aepfelbacher, 2003), and vesicle transport can be achieved rapidly and in less than 15 minutes, it may be possible that vesicle transport could facilitate the trafficking of podosome or invadopodia-associated proteins to the ventral membrane, which would allow construction of these structures and could offer a novel mechanism for the formation of an invasive structure.

## Invadopodia and Breast Cancer

Breast cancer cells will generate invadopodia in response to signals stimulated by growth factors, phorbol esters or interactions with the extracellular matrix (Yamaguchi et al. 2005; Yamaguchi and Condeelis, 2007). MDA-MB-231 breast carcinoma cells are an excellent system for studying invasion and metastasis and they will form invadopodia in response to stimuli. It is noteworthy that many proteins required for or associated with invadopodia formation are also associated with breast cancer progression, either through activation of signaling potential or changes in expression levels. In MDA-MB-231 cells, the expression levels and the signaling potential of the small GTP binding protein Arf6 was required for breast cancer invasion (Hashimoto et al. 2004; Onodera et al. 2005; Nam et al. 2007). Interestingly, Arf6 will relay signals from phorbol esters that promote phospholipase D activation and phosphatidic acid production, the latter of which is a component of vesicle membranes (Xu et al. 2003). Arf6 will couple with RalA, which can regulate the transport of vesicles to the ventral membrane (Caumont et al. 1998). Thus, it may be possible that Arf6 signaling is required for promoting phorbol ester or growth factor directed transport of vesicles to the ventral membrane that promote invadopodia formation. Another important signaling protein in breast cancer and invadopodia formation is cSrc, which exists on perinuclear vesicles and becomes activated upon trafficking to the membrane (Sandilands et al. 2004). cSrc is activated in breast cancer and will promote breast cancer formation in animal models. cSrc activation is a requirement for podosome and invadopodia formation (Linder, 2007). Indeed, the initial description of podosomes was associated with expression of the constitutively activated v-Src in fibroblasts (Tarone et al. 1985; Marchisio et al. 1988; Gavazzi et al. 1989). cSrc will phosphorylate a number of proteins on tyrosine and many of those substrates are relevant to breast cancer progression and are also found associated with both podosomes and invadopodia. Interestingly, phosphotyrosine signals will coalesce in podosomes and invadopodia (Kanner et al. 1991; Bowden et al. 2006). To this end, it is noteworthy that expression of the cSrc substrates cortactin and Tks5 are required for podosome formation (Seals et al. 2005; Webb et al. 2006). cSrc appears to play a role in podosome turnover and both the cSrc regulating protein CSK, as well as the tyrosine phosphatase PTP1B are required for podosome formation, likely by regulating dynamic changes in cSrc activity (Howell and Cooper, 1994; Cortesio et al. 2008). Each of these proteins are upregulated in breast cancer tissues and thus, could be well positioned to promote the formation of invasive structures and progression to an invasive phenotype. Thus, the protein components of podosomes and invadopodia may be very relevant to breast cancer.

## Podosome and Invadopodia Proteins May React to the Tumor Microenvironment

Other podosome and invadopodia associated proteins may also play important roles in the interpretation of outside-in signals that promote invasive potential. In the Met-1 breast cancer model system, the adhesion protein CD44 plays a role in invadopodia formation by linking ankyrin to the contractile actomyosin system (Bourguignon et al. 1998). In this regard, it may be possible that the adhesion aspects of podosomes, which do appear to differentiate them from invadopodia, could be regulated by contractile forces, much like focal adhesion plaques require negative contractile forces to promote adhesion (Dorfleutner et al. 2007). Studies in the MDA-MB-231 breast cancer cell line demonstrated that invadopodia will form in a stepwise fashion and promote invasive activity of breast carcinoma cells via expression of MT1-MMP (Kelly et al. 1998; Artym et al. 2006).

The ability of breast cancer cell lines to promote invasion and degradation of the extracellular matrix also correlated with an ability to phagocytose digested extracellular matrix proteins (Coopman et al. 1998). This function may be regulated by endophilin-2, SHIP-2, CIN85 and/or synaptojanin-2. SHIP-2 is an inositol 5-phosphatase found in podosomes, which removes the 5′ phosphate from phosphatidlyinositol-3,4,5-phosphate (Pesesse et al. 1998; Erneux et al. 1998). SHIP-2 is able to down regulate Fcγ-receptor mediated phagocytosis (independent of SHIP-1) and does this via an ability to down regulate Rac activity (Ai et al. 2006). Similarly, synaptojanin-2 is an inositol 5′-phosphatase found in invadopodia that regulates endocytic vesicle trafficking (Singer-Kruger et al. 1998). Synaptojanin-2 will bind to activated Rac and negatively regulate endocytosis (Malecz et al. 2000). Synaptojanin-2 is recruited to the membrane and stabilized by interactions with endophilin, which promotes clathrin-mediated endocytosis (Song and Zinsmaier, 2003). Interestingly, endophilin will also bridge interactions with dynamin 2 in podosomes (Ochoa et al. 2000) as well as with CIN85 (Petrelli et al. 2002). Here, a CIN85/endophilin complex was shown to affect changes in membrane curvature, which is consistent with a role for dynamin 2. Thus, the SHIP2 and/or synaptojanin-2/endophilin/dynamin-2/CIN85 proteins may play an important role in regulating the phagocytic activity associated with invasion by invadopodia as well as changes in membrane curvature that may promote vesicle trafficking or protease release. By this rationale, their appearance and association with invadopodia may be consistent with the function of these invasive structures. Further, it could be speculated that both invadopodia and podosomes utilize these signaling proteins in a similar manner to promote invasive potential. If true, then each of these proteins might be interesting drug targets that could be exploited to control breast cancer invasion.

## Summary

We have contrasted the differences and similarities between podosomes and invadopodia by cataloging the proteins found in these invasive structures and comparing their known and predicted functions for normal cells (podosomes) and carcinoma cells (invadopodia) in an effort to address the hypothesis that these two invasive structures may be related. To date, their is no evidence to indicate that invadopodia are derived from podosomes, or that each of these structures are derived from a common precursor structure. The major differences between the two are size, dependence on microtubular structures and subcellular localization upon the ventral membrane, whereby invadopodia are found below the Golgi bodies, while podosomes can be found either centrally located or at the leading edge of a migrating cell (Gimona et al. 2008). However, we speculate that given the common cellular signals that regulate their construction, common protein components and architecture, common size, shape and ventral membrane location, that these two structures are related. Further, a number of studies have shown the requirement for specific protein components in podosome and invadopodium formation and these same proteins are required for breast carcinoma cell invasion and are also expressed at high levels in breast cancer tissues. Probably the most interesting of these results were those done by Courtneidge and colleagues who have shown quite nicely the correlation between Tks5 in expression in breast cancer cells and its role in podosome formation and invasion (Seals et al. 2005). Future studies should focus on determining if theses structures are related and their role in breast cancer invasion, which will foster studies designed to create inhibitors that block invadopodia and podosome formation that may prevent breast carcinoma cells from invading.

## Figures and Tables

**Table 1.** Comparison of podosome and invadopodia associated proteins.

**Podosomes**	**Invadopodia**	**Function**
**Cytoskeletal components**		
Actin (Tarone et al. 1985)	Actin (Mueller et al. 1992)	Regulates cell contractility, motility and shape
Microtubules (Babb et al. 1997)	Unclear	Promote movement of motor proteins and vesicle transport
Intermediate Filaments (Correia et al. 1999)	Unclear	Cell shape and support
**Actin filament contractility**		
Tropomyosin 4 (Burgstaller and Gimona, 2004)	Unknown	Regulates actin filament contraction
Caldesmon (Eves et al. 2006)	Caldesmon (Yoshio et al. 2007)	Regulates actin filament contraction
Calmodulin (Eves et al. 2006)	Calmodulin (Bourguignon et al. 1998)	Ca^+2^ and actin filament binding protein that can affect contraction
Myosin IIA ((Burgstaller and Gimona, 2004; Kopp et al. 2006)	Myosin II (*implied in* (Bourguignon et al. 1998)	Binds actin filaments, provides contractile force
Calponin (Gimona et al. 2003)	Unknown	Ca^+2^ binding protein and regulator of myosin II function
**Actin filament cross linking**		
Sm22α (Transgelin) (Gimona et al. 2003)	Unknown	Regulates dynamic changes in actin filament cross linking and mesh-working
AFAP-110 (Gatesman et al. 2004)	AFAP-110 (Gatesman et al. 2004)	Regulates dynamic changes in actin filament cross linking and meshworking, src activating protein
Fimbrin (Messier et al. 1993; Babb et al. 1997)	Unknown	Actin filament cross linking protein
α-actinin (Chen, 1989)	α-actinin (Mueller et al. 1992)	Actin filament cross linking protein
Tensin (Hiura et al. 1995)	Tensin (Mueller et al.1992)	Actin filament cross linking protein
Palladin (Mykkanen et al. 2001)	Unknown	Actin filament cross linking, may link to VASP/mENA
**Actin filament branching**		
VASP (Mykkanen et al. 2001, 2001; Spinardi and Marchisio, 2006)	Unknown	Actin filament barbed end binding protein, promote motility, reduce Arp2/3 formation
Arp2/3 (Mizutani et al. 2002)	Arp2/3 (Yamaguchi et al. 2005)	Actin filament polymerization and branching
WASp (Calle et al. 2006; Chellaiah, 2006)	WASp (Desai et al. 2008)	Modulates actin filament polymerization
N-Wasp (Mizutani et al. 2002)	N-Wasp (Yamaguchi et al. 2005)	Modulates actin filament polymerization
WIP (Anton et al. 2007; Chabadel et al. 2007)	Unknown	Modulator of WASp and N-WASp function
HSP90 (Park et al. 2005)	Unknown	Chaperones N-WASP and regulates its ability to affect actin filament branching
CDC42 (Tatin et al. 2006; Moreau et al. 2006)	CDC42 (Furmaniak-Kazmierczak et al. 2007)	Affector of actin filament branching and polymerization via Arp2/3 and N-WASp, Small GTP binding protein, regulates filopodia formation
Cortactin (Webb et al. 2006)	Cortactin (Bowden et al. 1999)	Promotes actin filament polymerization and branching as an Arp2/3 modulator
**Actin filament severing/capping**		
Gelsolin (Biswas et al. 2004)	Unknown	Regulates actin filament severing and capping
Cofilin (Linder and Aepfelbacher, 2003)	Cofilin (Yamaguchi et al. 2005)	Regulates actin filament depolymerization and severing
Unknown	Nck (Yamaguchi et al. 2005)	Adaptor protein and regulator of actin filament polymerization
**Actin filament bridging**		
Talin (Marchisio et al. 1988)	Talin (Mueller et al. 1992)	Links integrins to actin filaments
Vinculin (Chen, 1989)	Vinculin (Mueller et al. 1992)	Links integrins to actin filaments
Zyxin (Spinardi and Marchisio, 2006)	Unknown	Actin scaffolding protein, biosensor that can modulate transcriptional changes in response to adhesion
Unknown	Ankyrin (Bourguignon et al. 1998)	Links actin filaments with integral membrane proteins
Kindlins (Ussar et al. 2006)	Unknown	Links actin filaments to membrane
**Intermediate Filaments**		
Vimentin (Correia et al. 1999)	Unknown	Intermediate filament protein, regulates positioning of organelles
**Microtubules**		
Kinesin-3 (Kopp et al. 2006)	Unknown	Motor protein, vesicle transport
**Cell Adhesion**		
β1, α3β1, α5β1, α6β1, αVβ3 Integrins (Marchisio et al. 1988; Spinardi and Marchisio, 2006; Calle et al. 2006)	β1, β3, αvβ3 Integrins (Deryugina et al. 2001) (Mueller et al. 1992; Nakahara et al. 1998)	Link cellular ventral membrane to the extracellular matrix
Unknown	Endoglin (Oxmann et al. 2008)	Transmembrane receptor part of TGFβ receptor complex and participates in cell adhesion
CD44 (Chabadel et al. 2007)	CD44 (Bourguignon et al. 1998)	Cell adhesion molecule that binds hyaluronic acid, MMP’s, collagen, osteopontin
**Adaptor Proteins**		
Paxillin (Spinardi and Marchisio, 2006;Calle et al. 2006)	Paxillin (Mueller et al. 1992)	Fak binding partner. Transcriptional activator.
p130cas (Honda et al. 1998; Yogo et al. 2006)	Unknown	Src binding partner. Required for transformation and podosome formation
Tks5/FISH (Abram et al. 2003)	Tks5/FISH (Seals et al. 2005)	5 SH3 domains, podosome ring protein
Eps8 (Goicoechea et al. 2006)	Unknown	Adaptor protein, binds receptors
Grb2 (Spinardi and Marchisio, 2006)	Unknown	Links to cell growth and RTK binding
Cbl (Bruzzaniti et al. 2005)	Cbl (Nam et al. 2007)	Adaptor, linked to ubiquitin machinery
STAT5 (Poincloux et al. 2007)	Unknown	Modulate transcription in response to cytosolic signaling
Calcitonin (Shyu et al. 2007)	Unknown	32 amino acid polypeptide that binds Ca^+2^ and reduces local Ca^+2^ levels
Caveolin 1 (Colonna and Podesta, 2005)	Unknown	Scaffolding protein, links integrins to tyrosine kinases, component of lipid rafts
**Tyrosine kinases**		
Src (Tarone et al. 1985)	Src (Chen, 1989)	PTK
Pyk2 (Chiusaroli et al. 2004)	Unknown	PTK, Fak-like
Csk (Howell and Cooper, 1994)	Unknown	Regulator of Src
Fak (Seals et al. 2005)	Fak (Hauck et al. 2002)	Integrin associated. Controversial association with invadopodia or podosomes
**Tyrosine Phosphatases**		
Unknown	PTP1B (Cortesio et al. 2008)	Regulator of cSrc
PTP epsilon (Chiusaroli et al. 2004)	Unknown	Regulator of cSrc
**Ser/thr kinases**		
Pak4 (Gringel et al. 2006)	Unknown	Effector of actin filament cross linking
Unknown	PKCmu (Bowden et al. 1999)	Effector of actin filament cross linking
Erk/Mek (Redondo-Munoz et al. 2006)	Erk/Mek (Furmaniak-Kazmierczak et al. 2007)	Effector of actin filament integrity
**Effectors of small GTP binding proteins and related signaling**		
αPIX (Gringel et al. 2006)	PIX (Furmaniak-Kazmierczak et al. 2007)	Pak binding partner and guanine nucleotide exchange factor (GEF)
ASAP1 (Bharti et al. 2007)	ASAP1 (Nam et al. 2007)	Arf GAP that uses lipids to become active (bind PH domain)
p190RhoGap (Burgstaller and Gimona, 2004)	p190RhoGap (Nakahara et al. 1998)	Negatively Regulates Rho function as a GAP
Unknown	Rock II (Vishnubhotla et al. 2007)	Positively Regulates Rho function
Dynamin2 (Ochoa et al. 2000)	Dynamin 2 (McNiven et al. 2004)	Affect vesicles and membrane invaginations that secrete MMPs, GTPase
Endophilin2 (Ochoa et al. 2000)	Unknown	Dynamin 2 and synaptojanin binding partner
**Lipid signaling**		
SHIP-2 (Yogo et al. 2006)	Unknown	Phosphoinositide 5′ phosphatase with SH2 domain
Unknown	Synaptojanin 2 (Chuang et al. 2004)	Phosphoinositide 5′-phosphatase, vesicle uncoating, effector of Rac1
CIN85 (Gaidos et al. 2007)	CIN85 (Nam et al. 2007)	Component of endocytic vesicles and binds Arf6 and ASAP1 (Arf6 GAP), associates with Cbl E3- ligase
**Proteases**		
MT1-MMP (Sato et al. 1997)	MT1-MMP (Chen and Wang, 1999)	Membrane bound metalloproteinase
ADAM12 (Abram et al. 2003)	Unknown	A type of MMP
MMP2 (Tatin et al. 2006)	MMP2 (Deryugina et al. 2002)	Soluble metalloproteinase, colla-genase and gelatinase
MMP9 (Linder, 2007)	MMP9 (Linder, 2007)	Soluble metalloproteinase, colla-genase and gelatinase
Calpain 2 (Calle et al. 2006)	Calpain 2 (Cortesio et al. 2008)	Ca^+2^ dependent cysteine protease
Unknown	Seprase (Ghersi et al. 2006)	Gelatinase and serine protease
Unknown	DPP4/CD26 (O’Brien and O’Connor, 2008)	Broad spectrum protease, degrades incretins
Unknown	uPAR (Kindzelskii et al. 2004)	Can link to integrins via UPARAP, binds uPA
Unknown	uPA (urokinase) (Kindzelskii et al. 2004)	Serine protease
Unknown	Type II serine protease (O’Brien and O’Connor, 2008)	Serine protease
Unknown	Invadolysin (likely) (McHugh et al. 2004)	Metalloprotease, cleaves lamin
Unknown	Legumain (Liu et al. 2003)	Cysteine protease

Unknown means unknown.
